# A *Drosophila* model of mitochondrial disease caused by a complex I mutation that uncouples proton pumping from electron transfer

**DOI:** 10.1242/dmm.015321

**Published:** 2014-08-01

**Authors:** Jonathon L. Burman, Leslie S. Itsara, Ernst-Bernhard Kayser, Wichit Suthammarak, Adrienne M. Wang, Matt Kaeberlein, Margaret M. Sedensky, Philip G. Morgan, Leo J. Pallanck

**Affiliations:** 1 Department of Genome Sciences, University of Washington, Seattle, WA 98195, USA.; 2 Molecular and Cellular Biology Program, University of Washington, Seattle, WA 98195, USA.; 3 Center for Developmental Therapeutics, Seattle Children’s Research Institute, Seattle, WA 98101, USA.; 4 Department of Pathology, University of Washington, Seattle, WA 98195, USA.

**Keywords:** Mitochondria, *Drosophila*, Mitochondrial disease, Respiratory chain, Leigh syndrome, Neurodegeneration

## Abstract

Mutations affecting mitochondrial complex I, a multi-subunit assembly that couples electron transfer to proton pumping, are the most frequent cause of heritable mitochondrial diseases. However, the mechanisms by which complex I dysfunction results in disease remain unclear. Here, we describe a *Drosophila* model of complex I deficiency caused by a homoplasmic mutation in the mitochondrial-DNA-encoded *NADH dehydrogenase subunit 2* (*ND2*) gene. We show that *ND2* mutants exhibit phenotypes that resemble symptoms of mitochondrial disease, including shortened lifespan, progressive neurodegeneration, diminished neural mitochondrial membrane potential and lower levels of neural ATP. Our biochemical studies of *ND2* mutants reveal that complex I is unable to efficiently couple electron transfer to proton pumping. Thus, our study provides evidence that the ND2 subunit participates directly in the proton pumping mechanism of complex I. Together, our findings support the model that diminished respiratory chain activity, and consequent energy deficiency, are responsible for the pathogenesis of complex-I-associated neurodegeneration.

## INTRODUCTION

Mitochondria perform numerous vital cellular functions, including producing the majority of ATP in cells, through oxidative phosphorylation via mitochondrial respiratory chain complexes. NADH ubiquinone oxidoreductase, or complex I, is the largest complex of the mitochondrial respiratory chain. It consists of ~45 subunits, including seven that are encoded by the mitochondrial genome: *NADH dehydrogenase subunit 1* (*ND1*)-*ND6* and *ND4L* (Efremov et al., 2010). All mitochondrial DNA (mtDNA)-encoded subunits of complex I span the mitochondrial inner membrane, where a subset of them might function in proton pumping given their homology to characterized bacterial complex I subunits (Amarneh and Vik, 2003; Dieteren et al., 2008; Efremov et al., 2010; Roberts and Hirst, 2012).

Mutations in genes encoding complex I subunits are a common cause of early-onset mitochondrial diseases such as Leigh syndrome, and mitochondrial myopathy, encephalomyopathy, lactic acidosis and stroke-like symptoms (MELAS) (Triepels et al., 2001). These diseases are highly debilitating, and are typically characterized by progressive neurodegeneration, seizures and shortened lifespan (Schon and Manfredi, 2003). However, the molecular mechanisms underlying the pathogenesis of diseases associated with complex I deficiency remain unclear (Torraco et al., 2009).

Recently, Xu and colleagues developed a novel technology for creating targeted mtDNA mutations in *Drosophila melanogaster*, and used this technology to generate strains that bear homoplasmic mtDNA mutations (Xu et al., 2008). Two of the strains that were generated using this approach harbor mutations in the *ND2* gene (Xu et al., 2008). Because mutations in human *ND2* have been demonstrated to cause Leigh syndrome (Ugalde et al., 2007), Leber’s hereditary optic neuropathy (Brown et al., 1992) and exercise intolerance (Schwartz and Vissing, 2002), we hypothesized that *Drosophila ND2* mutants might serve as an animal model of complex-I-associated disease. Here, we show that *ND2* mutants exhibit a variety of phenotypes that parallel symptoms of complex I deficiency in humans, including stress-induced seizures, progressive neurodegeneration and shortened lifespan. Moreover, our biochemical studies of *ND2* mutants reveal that their complex I inefficiently couples electron transfer to proton pumping, resulting in decreased mitochondrial membrane potential and diminished energy production. Our findings provide the first evidence that the ND2 subunit participates in the proton pumping activity of complex I, and suggest that symptoms associated with mitochondrial complex I deficiency derive from an energy deficit.

## RESULTS

### Drosophila ND2 mutants

In previous work, Xu and colleagues expressed a mitochondrially targeted restriction enzyme in the germline of *Drosophila* females to select for surviving offspring that bear an mtDNA mutation in the corresponding restriction site. Two of the strains that were generated using this approach harbor mutations in the *ND2* gene. One of these two mutants, *mt: ND2^ins1^* (*ND2^ins1^*), bears a three-nucleotide insertion in the *ND2* coding sequence, and the other, *mt: ND2^del1^* (*ND2^del1^*), bears a nine-nucleotide deletion in the *ND2* coding sequence (Xu et al., 2008). The *ND2^ins1^* mutation adds a serine at position 189, whereas the *ND2^del1^* mutation removes three amino acids at positions 186–188 of the ND2 protein (supplementary material Fig. S1). These mutations reside in evolutionarily conserved sequences (supplementary material Fig. S1), thus raising the possibility that flies bearing these mutations might serve as models of mitochondrial disease. Because the phenotypes of these fly strains were not characterized in previous work, we proceeded to test this hypothesis.

**TRANSLATIONAL IMPACT**

**Clinical issue**

Electron transport chain (ETC) complex I dysfunction is the most common cause of primary mitochondrial diseases. For example, mutations in the mitochondrial gene that encodes the complex I subunit NADH dehydrogenase subunit 2 (ND2) have been shown to be a cause of Leigh syndrome. Symptoms of Leigh syndrome include decreased lifespan, seizures, heat intolerance and neurodegeneration. Notably, individuals with Leigh syndrome can exhibit decreased complex I activity and abundance. However, the molecular mechanisms leading from ETC dysfunction to disease pathogenesis remain unclear.

**Results**

In this study, the authors characterize a *Drosophila melanogaster* model of complex I deficiency caused by a mutation in the mitochondrial-DNA-encoded gene *ND2*. The authors present biochemical and behavioral data showing that *ND2* mutants exhibit phenotypes that resemble Leigh syndrome. They report that these phenotypes include decreased lifespan, seizures, heat intolerance, movement deficits, neurodegeneration, and decreased complex I activity and abundance. In addition, the authors uncovered a defect in the ability of *ND2* mutants to pump protons through complex I. This defect was demonstrated by the observation of a decrease in the efficiency of complex I, but not complex II, to power ADP conversion to ATP in *ND2* mutants. The authors hypothesized that the pathogenesis of *ND2* mutants resulted from decreased efficiency of proton pumping by complex I, leading to energy deficits, and age-related neurodegeneration. Supporting this hypothesis, the authors demonstrated decreased ATP levels and decreased mitochondrial membrane potential in the brains of old *ND2* mutants.

**Implications and future directions**

Composed of 45 subunits, complex I is the most intricate complex of the ETC, but the functions of many of its subunits remain unclear. The authors’ findings show that *ND2* has a crucial role in the proton pumping mechanism of complex I, and point towards deficits in energy production as etiological factors underlying the pathogenesis of complex I deficiency. The *Drosophila* model described in this study exhibits a number of phenotypes amenable to genetic screening methodologies, and should therefore provide a powerful genetic system in which to further dissect the mechanisms underlying the pathogenesis of mitochondrial diseases.

### Behavioral analyses of *ND2* mutants

**Fig. 1.**
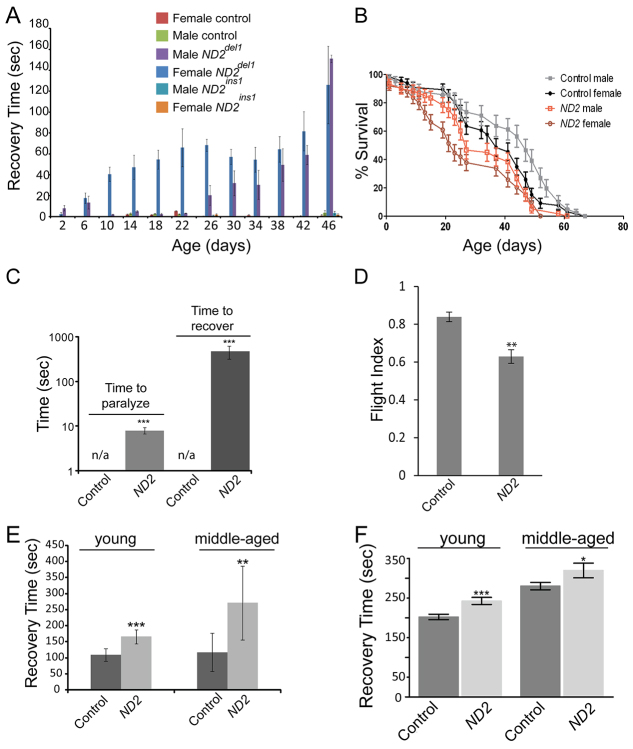
***ND2* mutants exhibit behavioral abnormalities and shortened lifespan.** (A) *ND2* mutants exhibit mechanical-stress-induced paralysis. Both male and female *ND2^del1^* and *ND2^ins1^* mutants, and male and female controls, were assayed for the length of time that flies remained paralyzed following mechanical stress. Error bars represent standard error of the mean (s.e.m.); *n*=3 independent groups of 6–12 individual animals. Measurements for *ND2^ins1^* mutants were taken at 2, 10, 26 and 46 days of age. (B) Lifespan analysis depicting a decrease in the median lifespan of *ND2* mutants. The median lifespan was 37 days for control females, 47 days for control males, 23 days for *ND2* mutant females and 27 days for *ND2* mutant males. Error bars represent s.e.m. (*P*<0.0001); *n*=10 independent groups of 12–20 animals for control and *ND2* mutants. (C) *ND2* mutants exhibit heat-induced paralysis. 14-day-old *ND2* mutants were incubated at 39°C, and the time for each individual fly to paralyze recorded in seconds. After 6 minutes, flies were placed in room-temperature vials, and the time to recover from paralysis recorded in seconds. Controls did not exhibit heat-sensitive paralysis over the time course of the assay (n/a). Histograms depict the median time to paralyze and recover from paralysis (seconds); *n*=3 independent groups of 7–9 animals; *P*=4.37×10^−17^ and *P*=8.7×10^−11^ for paralysis and recovery, respectively; ***P<0.001; error bars represent s.e.m. (D) *ND2* mutants have reduced flight ability. 7-day-old *ND2* mutants and controls were tested for their flying ability by measuring the distance that flies alighted when dispensed into a cylinder (*P*=0.001). Histograms represent the average flight index of the indicated genotypes, and error bars represent the s.e.m.; *n*=6 independent groups of 11–20 animals; **P<0.01. (E) *ND2* mutants are hypersensitive to CO_2_ exposure. Histograms depict the time to recover (seconds) for control and *ND2* mutant flies after hypercarbia-induced paralysis in young (3-day-old) and middle-aged (16-day-old) animals (*n*=2 groups of 12 animals; *P*=7.0×10^−5^ for 3-day-old animals; *n*=2 groups of 11 animals; *P*=0.0013 for 16-day-old animals; ***P≤0.001; **P<0.01); error bars represent standard deviations. (F) *ND2* mutants are hypersensitive to N_2_-induced hypoxia. Histograms depict the time to recover (seconds) for control and *ND2* mutant flies after hypoxia-induced paralysis in young (3-day-old) and middle-aged (16-day-old) animals (*n*=3 groups of 10–15 animals for each genotype); ***P=0.0004; *P=0.02; error bars represent the s.e.m.

Mitochondrial encephalopathies are typically characterized by neurological symptoms such as seizures (Pieczenik and Neustadt, 2007). Similarly, *Drosophila* strains with mutations affecting mitochondrial function often show an analogous seizure-like paralytic phenotype caused by mechanical stress, termed ‘bang sensitivity’ (Celotto et al., 2011; Fergestad et al., 2006; Ganetzky and Wu, 1982). To determine whether *ND2* mutants exhibited bang sensitivity, we subjected *w^1118^* (control), *ND2^del1^* and *ND2^ins1^* mutants to mechanical stress, and measured the time it took for them to recover from paralysis. Whereas control and *ND2^ins1^* mutants were unaffected by mechanical stress, *ND2^del1^* mutants displayed bang sensitivity that progressively worsened with age (Fig. 1A; supplementary material Movie 1). Our experiments also revealed that *ND2^del1^* mutant females displayed substantial bang-sensitive paralysis by 10 days of age, whereas *ND2^del1^* mutant males did not exhibit significant bang sensitivity until 26 days of age (Fig. 1A). Because *ND2^ins1^* mutants lacked a bang-sensitive phenotype, only *ND2^del1^* flies (hereafter referred to as *ND2* mutants) were used in our subsequent experiments.

The bang-sensitive phenotype of *ND2* mutants could be a consequence of reduced ND2 activity, or the acquisition of a novel activity. To distinguish between these possibilities, we tested whether restoring NADH dehydrogenase activity would rescue the bang-sensitive phenotype of *ND2* mutants. Previous studies have shown that ectopic expression of the yeast NADH dehydrogenase (*Ndi1*), which is composed of a single subunit, can rescue complex I deficiency in both mammalian cells and *Drosophila* (Cho et al., 2012; Seo et al., 1998). Thus, we tested whether expression of *Ndi1* could rescue the bang sensitivity of *ND2* mutants. *Ndi1* expression partially suppressed the bang-sensitive phenotype of *ND2* mutants, demonstrating that the *ND2^del1^* mutation results in a loss of ND2 activity (supplementary material Fig. S2A).

Mitochondrial diseases are often associated with dramatically shortened lifespan (Distelmaier et al., 2009; Ugalde et al., 2007). To determine if this was true for *ND2* mutants, we compared the lifespan of *ND2* mutants and controls. We found that the median lifespan of *ND2* mutants was reduced compared with wild-type controls, and that survival of female *ND2* mutants was shorter than that of male *ND2* mutants (Fig. 1B). Because female *ND2* mutants exhibited stronger phenotypes than males, only females were used in our subsequent experiments.

Another common symptom of mitochondrial disease is heat intolerance (Pieczenik and Neustadt, 2007). To test whether *ND2* mutants were sensitive to heat-induced paralysis, we exposed *ND2* mutants and controls to a temperature of 39°C, a condition previously shown to result in premature heat-induced paralysis in mitochondrial mutants (Ganetzky and Wu, 1982). We found that *ND2* mutants were fully paralyzed following exposure to 39°C for 6 minutes, and required a prolonged recovery period after being returned to room temperature. In contrast, age-matched controls failed to undergo heat-induced paralysis under these conditions (Fig. 1C).

Mitochondrial diseases preferentially affect tissues with high energetic demands, such as muscle and brain (Chinnery, 2000). To test for impaired function in muscle and/or neural tissue, we measured the flight performance of young *ND2* mutants using an established flight assay (Benzer, 1973; Greene et al., 2003). Young *ND2* mutants displayed significantly reduced flying ability compared with age-matched controls (Fig. 1D). In total, these experiments support the hypothesis that the *ND2^del1^* mutation affects energy-demanding muscle and/or brain tissue(s), mirroring deficits frequently seen in human mitochondrial disease.

Mitochondrial impairment has been shown to sensitize *Drosophila* to hypercarbia-induced (Whelan et al., 2010) and hypoxia-induced (Feala et al., 2007) paralysis, so we tested whether *ND2* mutants were also sensitive to these conditions. To induce paralysis, we exposed young and middle-aged *ND2* mutants and age-matched controls to carbon dioxide or nitrogen, respectively. We then measured the time required for flies to recover from paralysis upon return to ambient conditions. Both young and middle-aged *ND2* mutants took longer to recover from exposure to hypercarbic and hypoxic conditions than controls (Fig. 1E,F). Conversely, hyperoxic conditions have been shown to shorten wild-type *Drosophila* lifespan, and mitochondrial impairment enhances this effect (Whelan et al., 2010). To determine whether *ND2* mutants were sensitive to hyperoxic conditions, we housed young *ND2* mutants and controls in 100% oxygen and monitored their lifespan. As expected, *ND2* mutants and controls both exhibited a dramatically shortened lifespan under these conditions, but the *ND2* mutants had a significantly shorter lifespan relative to controls (supplementary material Fig. S2B).

### Tissue integrity of *ND2* mutants

**Fig. 2.**
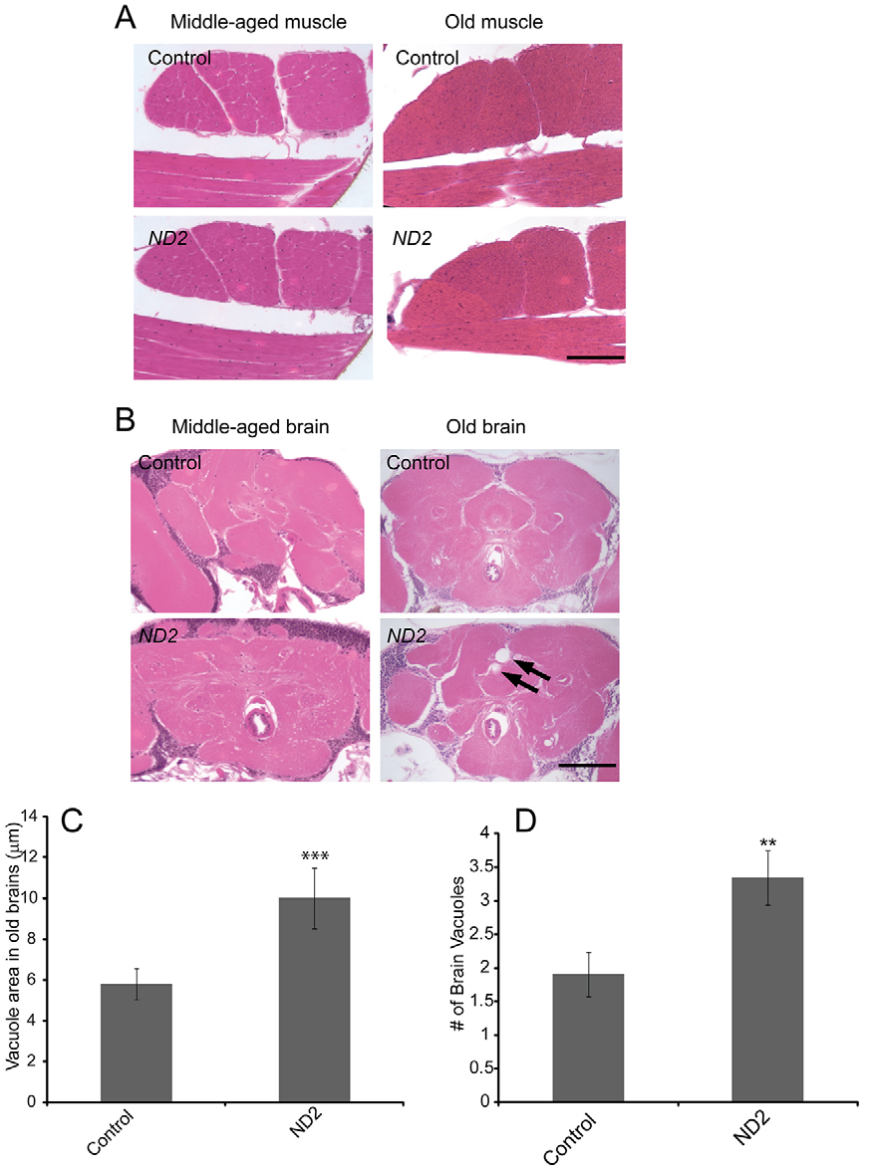
***ND2* mutants exhibit progressive neurodegeneration, but lack muscle pathology.** (A) Thoracic muscle integrity was analyzed by coronal sectioning of the thorax of middle-aged (26-day-old) or old (42-day-old) *ND2* mutants and controls. Representative images of hematoxylin- and eosin-stained thoracic muscle sections are depicted. (B) Brain integrity was analyzed by coronal sectioning of the heads of middle-aged or old *ND2* mutants and controls. Representative images of hematoxylin- and eosin-stained coronal brain sections are depicted. Arrows demark neurodegenerative vacuoles. Scale bars: 200 μm. (C) Quantification of brain vacuole size demonstrates an increase in vacuole size in *ND2* mutants (*P*=4×10e^−5^). Vacuoles were rarely detected in the brains of middle-aged *ND2* mutants and were, therefore, not subjected to quantification. Error bars represent s.e.m.; *n*=26 animals for *ND2* mutants; *n*=21 animals for controls; ***P<0.001. (D) Quantification of the number of brain vacuoles demonstrates an increase in vacuole number in *ND2* mutants (*P*=0.008). Vacuoles were rarely detected in the brains of middle-aged control or *ND2* mutants and were, therefore, not subjected to quantification. Error bars represent s.e.m.; *n*=26 animals for *ND2* mutants; *n*=21 animals for controls; **P<0.01.

A hallmark of mitochondrial disease is the progressive degeneration of muscle and/or neural tissue(s) (Finsterer, 2008). We therefore analyzed muscle and brain integrity by hematoxylin and eosin staining of tissue from middle-aged and old *ND2* mutants and controls. These analyses revealed no abnormalities in the flight muscles of middle-aged or old *ND2* mutants or in the brains of middle-aged *ND2* mutants (Fig. 2A,B). However, neurodegenerative vacuoles observed in the brains of old *ND2* mutants were both significantly larger and more numerous than those seen in age-matched controls (Fig. 2B–D), indicating that the mutants have a progressive neurodegenerative phenotype.

To test whether *ND2* mutants have subtle defects in muscle integrity that were not evident from our histological analyses, we used confocal microscopy to compare cytoskeletal architecture, mitochondrial morphology and apoptotic cell death in the indirect flight muscles of *ND2* mutants and controls by staining for actin, cytochrome c and cleaved caspase-3, respectively. We also included *parkin*-null animals in these studies as a positive control because they have previously been shown to exhibit apoptotic degeneration of indirect flight muscles, including disruption of actin organization, and altered mitochondrial morphology (Greene et al., 2003; Klein et al., 2014). We did not detect significant differences in actin organization, mitochondrial size or cleaved caspase-3 staining between *ND2* mutants and aged-matched controls (supplementary material Fig. S3), thus providing further evidence that the *ND2^del1^* mutation does not affect the musculature. However, in agreement with previous work, we observed disruption of the actin cytoskeleton, enlarged mitochondria and cleaved-caspase-3 staining in *parkin*-null flight muscle preparations (supplementary material Fig. S3).

### Functional analyses of mitochondria isolated from *ND2* mutants

**Fig. 3.**
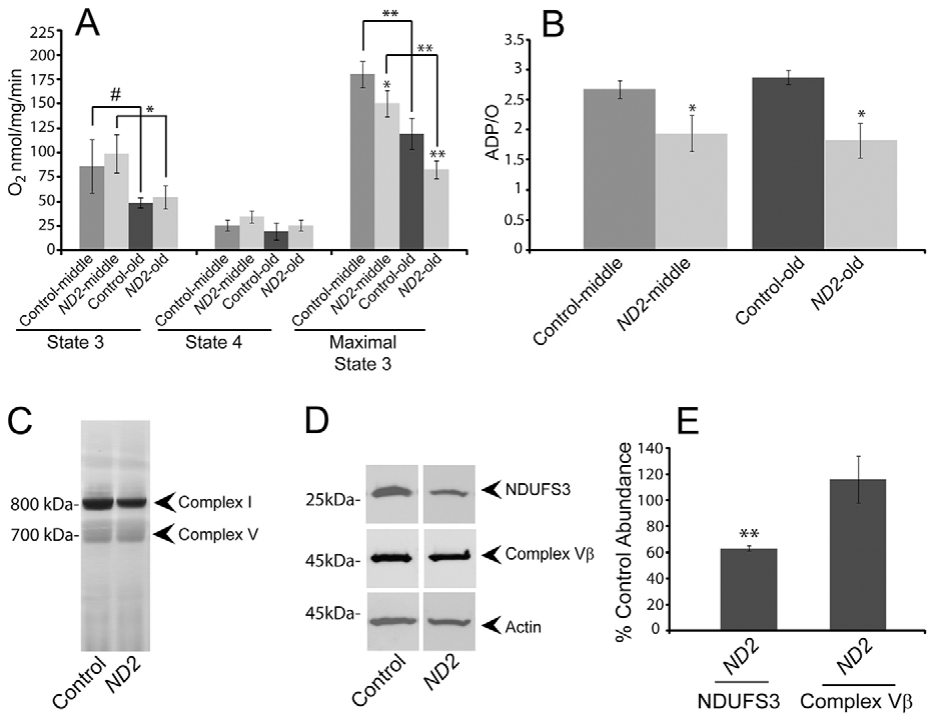
***ND2* mutants exhibit decreased mitochondrial function.** (A) Mitochondrial complex-I-dependent respiration rates were measured in mitochondria isolated from middle-aged (14-day-old) or old (30- to 35-day-old) *ND2* mutant or controls, revealing age-related declines in both state 3 and maximal state 3 values within genotypes (*P*=0.08 and *P*=0.03 for comparison of middle-aged and old control or *ND2* mutant state 3 values, respectively; *P*=0.001 and *P*=0.003 for comparison of middle-aged and old control or *ND2* mutant maximal state 3 values, respectively). State 4 respiration was similar under all conditions tested. No differences were found for state 3 values between control and *ND2* mutants at either middle or old ages. However, maximal state 3 values were decreased in both middle-aged and old *ND2* mutants when compared with age-matched controls (*P*=0.04 for middle-aged and *P*=0.003 for old animals). Error bars represent standard deviations; *n*=3 independent mitochondrial preparations; **P<0.01; *P<0.05; ^#^*P*<0.1. (B) Oxidative phosphorylation efficiency (ADP/O) is decreased in *ND2* mutants relative to controls at both middle and old ages (*P*=0.02 and *P*=0.01, respectively). Error bars represent standard deviations; *n*=3 independent mitochondrial preparations; *P<0.05. (C) Blue native gel/complex I in-gel activity analysis of mitochondria isolated from middle-aged (14-day-old) *ND2* mutants and controls reveals similar levels of complex V in both genotypes, but decreased fully assembled complex I abundance in *ND2* mutants. (D) Western blot analysis of protein extracts from 14-day-old *ND2* mutants and controls reveals a specific decrease in the abundance of the complex I subunit NDUFS3, with no decrease in the complex Vβ subunit. (E) Quantification of western blot data for NDUFS3 (*n*=3 independent tissue extracts; *P*=0.003) and complex Vβ (*n*=3 independent tissue extracts; *P*=0.46) levels normalized to actin, and expressed as the % of control values. Error bars represent s.e.m.; **P<0.01.

To explore the biochemical mechanisms underlying the *ND2* mutant phenotypes, we isolated mitochondria from middle-aged and old *ND2* mutants and controls, and used complex-I-specific substrates to measure mitochondrial respiration. We performed these experiments using both limiting and saturating amounts of ADP to measure state 3 (ADP-stimulated) and maximal state 3 complex-I-dependent respiratory rates, respectively (supplementary material Fig. S4). As previously reported, state 3 and maximal state 3 rates decreased with age in control flies (Ferguson et al., 2005), and *ND2* mutants displayed similar kinetics of decreased activity with age (Fig. 3A). Mitochondria isolated from either middle-aged or old *ND2* mutants showed unimpaired state 3 respiration, but maximal state 3 values were significantly decreased in *ND2* mutants relative to age-matched controls (Fig. 3A and Table 1). The *ND2^del1^* mutation thus causes a complex-I-dependent respiratory defect specifically under maximally demanding conditions. To ensure that there was no functional variability between mitochondrial preparations caused by our isolation method, we measured complex-II- and complex-IV-dependent respiration in *ND2* mutants and controls. We found no significant difference in complex-II-dependent respiration, complex-II-dependent ADP/O ratios or complex-IV-dependent respiration between *ND2* mutant and control mitochondria (Table 1). These results confirm the integrity of our mitochondrial preparations, and the respiratory comparisons made between *ND2* mutants and controls (Fig. 3A).

**Table 1.**
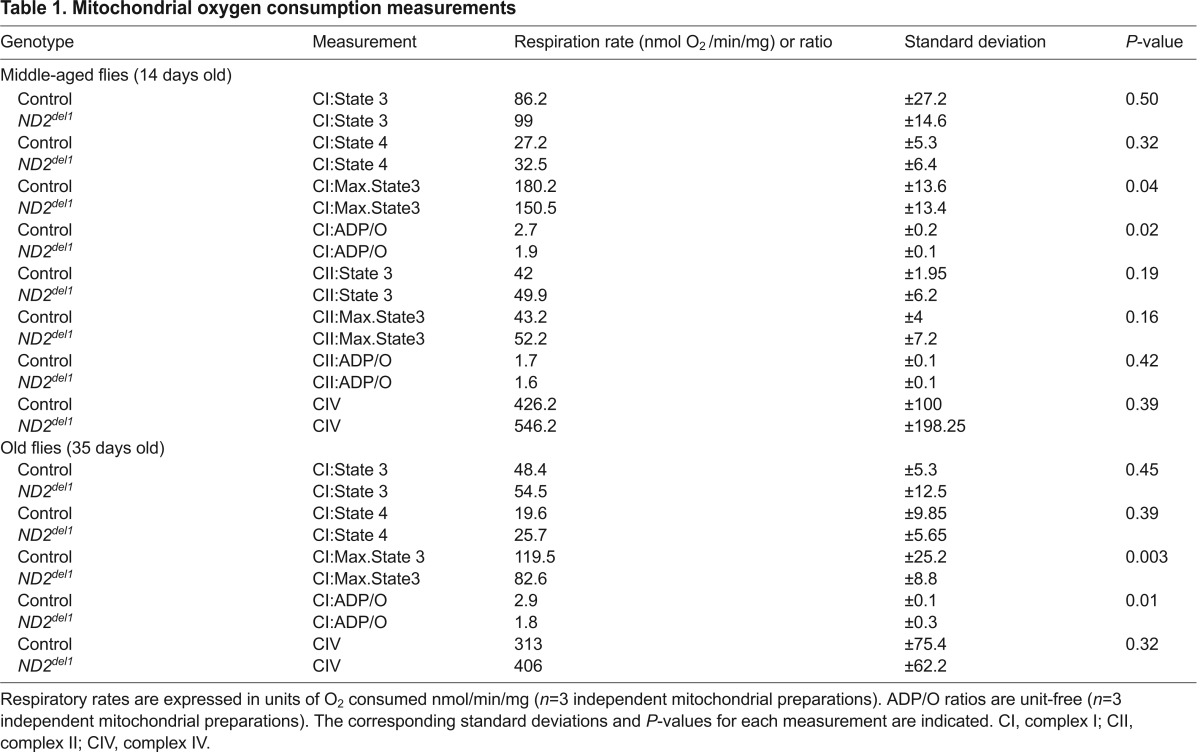
Mitochondrial oxygen consumption measurements

### Oxidative phosphorylation coupling

The respiratory defect of *ND2* mutants offered the opportunity to investigate the functional role of the ND2 subunit in complex I activity. To test whether ND2 plays a role in the proton pumping activity of complex I, we measured the linkage of electron transport to ATP production by determining the ADP/O ratio. Because electron transport is mechanistically coupled to ADP phosphorylation via the proton circuit across the inner membrane, any defect that reduces the efficiency of complex I in pumping protons should lead to a decrease in ADP/O. We found a significant decrease in complex-I-dependent ADP/O ratios in both middle-aged and old *ND2* mutants (Fig. 3B and Table 1). This decrease is likely not explained by a change in the efficiency of ATP production by complex V, nor a proton leak in the mitochondrial membrane, because *ND2* mutant ADP/O ratios were normal when respiration was dependent on complex II (Table 1). Moreover, the mitochondrial respiratory rate following depletion of ADP (state 4), an indicator of proton leak across the mitochondrial inner membrane, was unaffected by the *ND2^del1^* mutation (Fig. 3A and Table 1). Thus, our results demonstrate that electron flow through complex I is inefficiently coupled to proton pumping in *ND2* mutants, indicating that the *ND2* subunit functions in the proton pumping mechanism of complex I.

### Complex I integrity

Although the decrease in ADP/O in *ND2* mutants indicates that ND2 is required for efficient coupling of electron transfer to proton pumping, it does not explain the decrease of maximal complex-I-dependent state-3 respiration in *ND2* mutants. One possible explanation for this decrease is a reduction in complex I abundance. To test this model, we examined mitochondria isolated from *ND2* mutants and controls using blue native gels. This analysis revealed that the abundance of fully assembled complex I was decreased in *ND2* mutants relative to controls, without an accompanying decrease in complex V abundance (Fig. 3C). Western blot analysis also revealed a decrease in the abundance of the complex I subunit NDUFS3 in *ND2* mutants, but little to no change in the abundance of the β-subunit of complex V relative to age-matched controls (Fig. 3D,E and supplementary material Fig. S5). These findings suggest that ND2 is also required for the structural integrity of complex I.

### Mitochondrial membrane potential and ATP production

**Fig. 4.**
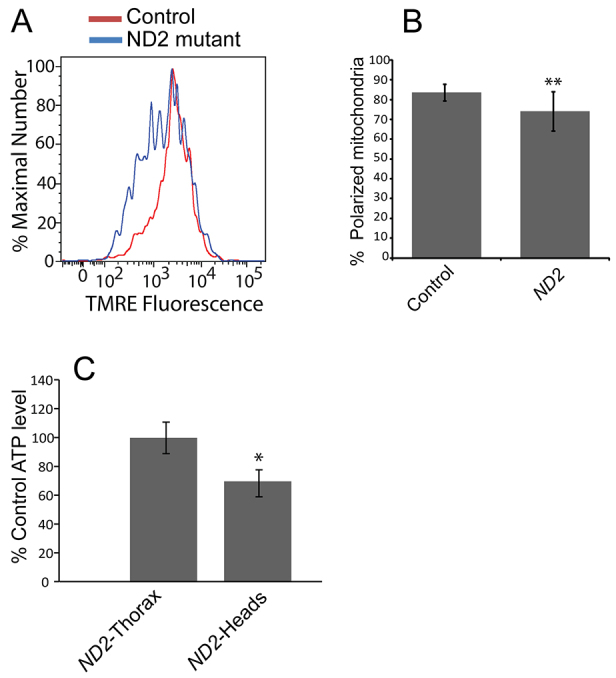
***ND2* mutants exhibit decreased mitochondrial membrane potential and ATP production.** (A) Neurons from 30- to 35-day-old *ND2* mutants and controls labeled with the mitochondrial membrane potential dye TMRE were analyzed by flow cytometry. Histograms depict the percent maximal number of cells measured at each TMRE fluorescence intensity. (B) Quantification of the average percent of isolated neurons exhibiting mitochondrial membrane-potential-dependent fluorescence above 10^3^ arbitrary units (% polarized mitochondria) is shown for control and *ND2* mutants (*P*=0.004). Error bars represent standard deviation; *n*=3 independent brain preparations; **P<0.01. (C) ATP levels were determined in heads of 35-day-old *ND2* mutants and controls, and displayed relative to control values (*P*=0.05). Error bars represent s.e.m.; *n*=3 groups of 5 individuals; *P≤0.05 for a one-tailed *t*-test.

The decreased maximal complex-I-dependent state-3 activity and ADP/O ratio of *ND2* mutants suggested that the mutants might have diminished mitochondrial membrane potential and consequently reduced ATP synthesis. To test these possibilities, we measured mitochondrial membrane potential in dissociated neurons from old *ND2* mutants and controls, and we compared ATP levels in the thoraces and heads of old *ND2* mutants and controls. We detected a decrease in the fraction of cells with polarized mitochondria in the brains of old *ND2* mutants relative to controls, and reduced ATP abundance in *ND2* mutants that was specific to heads (Fig. 4A–C). These findings suggest that the *ND2^del1^* mutation affects the proton pumping activity of complex I, which reduces the proton gradient available for ATP generation, leading to energy deficiency in the brains of *ND2* mutants.

### Reactive oxygen species damage

Mitochondrial complex I deficiency is believed to trigger the formation of reactive oxygen species (ROS), and increased ROS production is a widely believed cause of the symptoms accompanying complex-I-associated diseases (Lin et al., 2012). To test whether the phenotypes of *ND2* mutants could derive from excessive ROS production, we measured the abundance of a common byproduct of oxidative stress: 4-hydroxynonenal (4-HNE)-containing protein adducts. Levels of 4-HNE protein adducts are elevated under conditions of increased oxidative stress (Poli and Schaur, 2000), and their abundance can be easily measured using an antiserum that detects 4-HNE adducts, as has been previously shown in *Drosophila* (Sun et al., 2014; Tsai et al., 1998). The use of this antiserum on a western blot of protein extracts from *ND2* mutants and age-matched controls revealed no significant alteration in the abundance of 4-HNE protein adducts between these genotypes in young, middle-aged or old animals (supplementary material Fig. S6). These data argue that the phenotypes of *ND2* mutants are not a consequence of increased ROS-mediated damage.

## DISCUSSION

A number of hypotheses have been proposed to explain the pathological consequences of complex I deficiency. Among the most enduring of these models are that complex I deficiency results in the elevated production of toxic ROS and/or an energy deficit (Torraco et al., 2009; Lin et al., 2012). To address these and other possible models of pathogenesis, we studied a *Drosophila* strain with a mutation affecting the mitochondrial-encoded respiratory chain complex I subunit ND2. We show that *ND2* mutants exhibit many of the behavioral and histological characteristics of mitochondrial disease, suggesting that *ND2* mutants represent a valid model of this spectrum of disorders. Moreover, our work shows that *ND2* mutants have diminished ATP abundance, mitochondrial membrane potential, complex I abundance, and complex I activity. However, we detected no evidence of increased ROS in *ND2* mutants. These findings support a model in which the pathogenesis accompanying complex I deficiency is caused by diminished energy production.

Several of our findings contrast with previously published work using other models of complex I deficiency. For example, studies of *Ndufs4* knockout mice, and transgenic mice harboring a pathogenic point mutation in *ND6*, revealed decreased maximal complex I activity, and increased levels of ROS-mediated damage (Quintana et al., 2010; Lin et al., 2012). These studies suggest that ROS is a contributor to the pathogenesis of complex I deficiency. However, ROS levels are thought to increase as a result of decreased basal complex I activity (Turrens, 2003). Therefore, it is possible that *ND2* mutants do not show changes in ROS-mediated damage because of their normal basal complex I activity, whereas the *Ndufs4* and *ND6* mouse models might exhibit increased ROS-mediated damage as a result of decreased basal complex I activity. Further analyses of the basal activity of complex I in these mouse models of complex I deficiency will be required to test this hypothesis. Another discrepancy of our work concerns the mild phenotypes of *ND2* mutants relative to other fly and mouse models of complex I deficiency. We believe this discrepancy is best explained by differences in the severity of the mutations analyzed in each respective study. The *ND2^del1^* allele is an in-frame deletion predicted to make a full-length protein lacking three amino acids. This mutation could therefore represent a hypomorphic allele of *ND2*. By contrast, many animal models of complex I deficiency, with phenotypes more severe than those of *ND2* mutants, harbor severe loss-of-function or null alleles (Cho et al., 2012; Kruse et al., 2008; Zhang et al., 2013). In further support of this model, the magnitudes of the decreases in complex I activity and abundance in *ND2^del1^* mutants are similar to those seen in *Caenorhabditis elegans* with partial inactivation of complex I subunits (Falk et al., 2009). The relatively mild phenotypes of *ND2* mutants potentially make them well-suited to genetic modifier screens aimed at the identification of currently unknown genetic factors that influence the phenotypes associated with complex I deficiency.

In addition to exploring the pathogenic mechanisms underlying complex I deficiency, our work provides insight into the role of ND2 in complex I function. Although the ND2 subunit has been hypothesized to pump protons based on its membrane localization and sequence homology to a family of cation antiporters (Efremov et al., 2010), only a study of the bacterial ND2 homolog, NuoN, has provided support for this model (Amarneh and Vik, 2003). Our finding that the *ND2^del1^* mutation results in uncoupling of proton pumping from electron transfer [i.e. decreased oxidative phosphorylation efficiency (ADP/O) with normal basal complex-I-mediated electron flux] strongly supports a direct role for ND2 in the proton pumping mechanism of eukaryotic complex I.

Although our work advances the understanding of the consequences of ND2 deficiency, and the functional role of the ND2 subunit in eukaryotes, it also raises several questions. One important question raised by our work concerns the mechanisms underlying the progressive nature of the *ND2* mutant phenotypes. One possible explanation of the progressive phenotypes of *ND2* mutants is that energy deficiency does not occur until a critical threshold of complex I activity is crossed. As previously reported (Ferguson et al., 2005), and confirmed in our current study, complex I activity naturally declines with age in *Drosophila*. This decline in complex I activity might not reach the point where it results in an energy deficiency over the natural lifespan of *Drosophila*. However, the ND2 mutation exacerbates this natural decline in complex I activity, and thus could account for the progressive nature of the *ND2* mutant phenotypes.

Another important question raised by our study is why a systemic deficit in complex I function results in selective neurodegeneration. One possible explanation could be that neurons are more sensitive to loss of mitochondrial function because they rely primarily on oxidative phosphorylation for ATP, whereas other tissues, such as muscle, are capable of using both oxidative phosphorylation and glycolysis (Chan et al., 2009). Although neural selective vulnerability is not uncommon in mitochondrial-associated diseases, including Leigh syndrome, Leber’s hereditary optic neuropathy and Alpers’ syndrome (Bianchi et al., 2011; Koopman et al., 2013), further studies will be required to understand the mechanisms underlying the progressive tissue-specific nature of mitochondrial disease.

In summary, our work suggests an important role for ND2 in the proton pumping mechanism of complex I, and provides a genetically tractable animal model of complex I deficiency. This model should prove a valuable tool in which to further explore the pathological mechanisms underlying human mitochondrial disease.

## MATERIALS AND METHODS

### *Drosophila* strains and maintenance

All *Drosophila* strains were maintained on standard cornmeal/molasses medium at 25°C with a 12-hour light-dark cycle. The *ND2^del1^* and *ND2^ins1^* stock was obtained from the laboratory of Dr Patrick O’Farrell (University of California, San Francisco, CA) (Xu et al., 2008). The isogenic *w^1118^* stock was obtained from the Bloomington *Drosophila* Stock Center at Indiana University. To control for differences in nuclear genetic background, we outcrossed *ND2* mutants to the *w^1118^* strain. F_1_ offspring derived from crossing *ND2* mutant females to *w^1118^* males were used as the experimental group, given that they inherit mtDNA from the *ND2* mutant strain; F_1_ offspring derived from crossing *ND2* mutant males to *w^1118^* females were used as the control group, given that they inherit mtDNA from the *w^1118^* strain. The homoplasmic status of the *ND2^del1^* mutation from the outcrossed *ND2* mutant strain was reconfirmed by PCR and restriction digest analysis (data not shown). *Parkin*-null animals harbored the previously described *park^25^* null allele (Greene et al., 2003), and were composed of the genotype *CyO/IF; park^25^/park^25^*. Previously described transgenic flies capable of expressing yeast Ndi1 were obtained from Dr David Walker (Cho et al., 2012).

### Mechanical-stress-induced paralysis

Flies were assayed for bang sensitivity using a modification of a previously published protocol (Ganetzky and Wu, 1982). Briefly, flies were vortexed at maximum speed for 10 seconds in inverted glass vials containing cotton stoppers, and the time required for each individual animal to right itself was recorded (supplementary material Movie 1).

### Heat-induced paralysis

14-day-old flies were assayed for heat-induced paralysis by placing groups of five to ten animals into pre-warmed vials maintained at 39°C. The time for the flies to become paralyzed was recorded. After exposure to 39°C for 6 minutes the animals were then placed in new room-temperature vials (~20°C), and the recovery time of *ND2* mutants from paralysis recorded.

### Lifespan

Lifespan assays were performed using vials containing 12–20 flies. Flies were transferred to new vials every 2 days throughout the assay period. At least 60 flies were monitored for each experiment. For sex-specific lifespan assays, *ND2* mutant males and females or gender- and age-matched control flies were separated within 1 day of eclosion, and the assay conducted as described above.

### Flight

Flight assays were performed as previously described (Benzer, 1973; Greene et al., 2003). Briefly, an acetate sheet was divided into five parts, coated with vacuum grease and inserted into a 1 liter graduated cylinder. 7-day-old flies were gently tapped into a funnel at the top of the cylinder, and became stuck to the vacuum grease where they alighted. The acetate sheet was removed, and the number of flies in each section was counted. Flies that alighted in a higher section of the acetate sheet received a correspondingly higher value in scoring. The number of flies alighting in the top section was summed and multiplied by four, the number of flies alighting in the second highest section was summed and multiplied by three, and so on. Finally, the weighted sum for the entire group of flies was determined, and normalized to four times the total number of flies used in the assay (the maximum possible score), and this normalized value was reported as the flight index. Flies that alighted at the top of the cylinder received a flight index score of 1, whereas flies that fell to the bottom of the cylinder received a flight index score of 0. At least six groups of 11–20 flies per genotype were tested.

### Sensitivity to CO_2_ exposure

Young (3-day-old) or middle-aged (16-day-old) control or *ND2* mutants were exposed to 100% CO_2_ (Praxair) on a gas-permeable mesh pad for 1 minute, rendering them completely immobile. The gas was then shut off, leaving the flies exposed only to room air, and the time to recover from paralysis was measured.

### Hypoxia sensitivity

Three separate sets of 10–15 age-matched young (3-day-old) and middle-aged (16-day-old) flies were placed into 70 ml chambers and 100% N_2_ (Praxair) certified at less than 10 ppm O_2_ was flowed through the chamber at 350 ml/minute for 1.5 minutes to induce paralysis. After 30 seconds, flies were exposed to room air at the same rate, and the time from the introduction of room air to recovery from paralysis was recorded.

### Hyperoxia sensitivity

Female flies were collected within 48 hours of eclosion and placed into four vials of 25 flies per genotype. Vials were then placed into a temperature-controlled hyperoxia chamber and maintained in a 100% O_2_ environment at 25°C. Flies were transferred to new vials every 24 hours and scored for survival.

### Confocal microscopy

Indirect flight muscles were dissected from 40-day-old control or *ND2* mutant animals, or from 7-day-old *parkin*-null animals in phosphate buffered saline (PBS). Dissected muscles were placed in 0.3% Triton X-100 (Sigma), 4% paraformaldehyde (Ted Pella Inc.) in PBS, and rotated at room temperature for 40 minutes. Muscles were then washed twice in PBS and placed in blocking buffer [0.3% Triton X-100, 5% normal goat serum (Fisher Scientific) in PBS] for 45 minutes. Muscles were then incubated with a mouse primary antibody raised against cytochrome c (BD Labs #556433) (1/1000), and a rabbit primary antibody raised against cleaved caspase-3 (Cell Signaling #9661) (1/400) for 2 days at 4°C in 0.3% Triton X-100 and 2.5% normal goat serum in PBS. Tissue was then washed twice in 0.3% Triton X-100 in PBS, for 20 minutes each, at room temperature. The tissue was then placed in secondary antibodies: mouse-Alexa-Fluor-647 and rabbit-Alexa-Fluor-488 (Molecular Probes), and incubated for 1 hour rotating at room temperature. At 20 minutes prior to removal from secondary antibodies, phalloidin-568 (Life Technologies) was added at a concentration of 1/400. Tissue was removed and washed two times for 10 minutes each in 0.3% Triton X-100 in PBS. The muscles were then placed in PBS containing DAPI (Sigma-Aldrich) at 2 μg/ml for 5 minutes. Cells were washed in PBS for 10 minutes, mounted between two glass slides with Fluoromount (Sigma-Aldrich), and imaged using an Olympus FV-1000 with a 60× lens and a 4× digital zoom.

### Image quantification

Images of muscles stained with cytochrome c were opened in ImageJ, converted to 8-bit, thresholded, watershed, and analyzed using the analyze particle tool. The average perimeter of distinct mitochondria was used as an indicator of mitochondrial morphology and mass. For cleaved-caspase-3 staining, the number of cleaved-caspase-3-positive myocytes, as indicated by DAPI-positive/cytochrome c/cleaved-caspase-3-positive staining, were manually counted.

### Tissue preparation and sectioning

Middle-aged (14-day-old) or old (42-day-old) flies were anesthetized, mounted in fixing collars and placed in Carnoy’s fixation solution (10% acetic acid; 30% chloroform; 60% absolute ethanol) for 3.5 hours. Fixed flies were then placed in 95% ethanol twice for 30 minutes each, in 100% ethanol for 45 minutes, then in methyl benzoate overnight. Flies were then incubated in a 1:1 mixture of methyl benzoate:paraffin for 1 hour at 60°C, transferred to 100% liquid paraffin and incubated at 60°C for 30 minutes, then transferred to fresh 100% liquid paraffin four times for 15–30 minutes each. The flies were next transferred to 100% liquid paraffin in a plastic mold, placed at 60°C for 15 minutes, and the paraffin was allowed to harden at room temperature. 5-μm-thick coronal histological sections were cut on a Shandon Finesse 325 Microtome, and processed for hematoxylin and eosin staining. Images were collected on a Nikon Optiphot-2 using a 20× objective. Vacuole size was quantified in ImageJ (Abramoff et al., 2004) by converting pixel values into micrometers (0.439 μm/pixel). Only vacuoles present in at least two consecutive sections were quantified to avoid potential artifacts derived from tissue sectioning.

### Mitochondrial preparations

Mitochondria were obtained from 0.25–0.5 g of 14-day-old or 30- to 35-day-old flies. Flies were chilled on ice, and then homogenized using a chilled Potter/Elvehjem homogenizer with 10 manual strokes in 20 ml of homogenization buffer (200 mM mannitol, 70 mM sucrose, 5 mM MOPS, 2 mM EDTA; 0.4% defatted BSA; pH 7.4), avoiding shearing. The homogenate was then filtered through cotton gauze, and spun at 300 ***g*** for 4 minutes at 4°C. The supernatant was then filtered through gauze and spun at 10,000 ***g*** for 10 minutes at 4°C. The pellet was resuspended in 10 ml of resuspension buffer (200 mM mannitol, 70 mM sucrose, 5 mM MOPS, 2 mM EDTA; pH 7.4), and spun at 10,000 ***g*** for 10 minutes at 4°C. The mitochondrial pellet was then resuspended in 10–50 μl of final buffer (200 mM mannitol, 70 mM sucrose, 5 mM MOPS; pH 7.4), to a final concentration of ~50 mg protein/ml. 150–300 mg of mitochondria was used for oxidative phosphorylation assays, and 250 mg for blue native gel analysis. All reagents were obtained from Sigma (St Louis, MO).

### Oxidative phosphorylation assays

Oxygen consumption of isolated mitochondria was monitored using a Clark-type electrode (Oxytherm with analysis software Oxyg32, Hansatech Instruments, Pentney, Norfolk, UK) by an adaptation of previous methods (Kayser et al., 2001). Briefly, 150–300 μg of purified mitochondria were stirred in 500 μl of assay buffer (100 mM KCl, 50 mM MOPS, 1 mM EGTA, 5 mM potassium phosphate, 1 mg/ml defatted BSA; pH 7.4) at 30°C. 10 mM malate and 20 mM pyruvate were added as complex-I-specific electron donor substrates, 20 mM succinate was added as a complex-II-specific electron donor substrate or 25 mM TMPD and 250 mM ascorbate (pH 7.0) were added as complex-IV-specific electron donor substrates. A low dose of ADP (90.7 nmol) was added to determine state 3 mitochondrial respiration rates and, following depletion of ADP, ensuing state 4 respiration rates. A saturating dose of ADP (1 μmol) was used to assess maximal complex-I-dependent electron transport capacity under phosphorylating conditions (maximal state 3). Saturating amounts of TMPD/ascorbate were used to measure complex IV activity. ADP/O was defined as the number of ADP molecules phosphorylated per oxygen atom reduced to water, and ADP/O was calculated by dividing the amount of ADP added (90.7 nmol) by the amount of oxygen consumed between the time of ADP addition and the onset of state 4 (supplementary material Fig. S4).

### ATP determination

Heads or thoraces were sectioned from five 35-day-old flies and homogenized in 6 M guanidine HCl (Sigma); 10 mM TRIS (Sigma), pH 7.3, as previously described (Xu et al., 2008). ATP content was measured using an ATP determination kit (Molecular Probes, Eugene, OR). Protein abundance was measured using a Bradford protein assay, and ATP abundance was normalized to total protein abundance as previously described (Xu et al., 2008).

### Mitochondrial membrane potential

Measurement of neural mitochondrial membrane potential from 30- to 35-day-old flies was conducted as previously described (Burman et al., 2012), except that dissections were performed in supplemented DME/Ham’s F-12 High Glucose media lacking phenol red (Sigma), and all incubation steps were carried out at 25°C. In addition, 10 nM tetramethylrhodamine ethyl ester (TMRE) was utilized throughout the protocol. The % of cells harboring polarized mitochondria was determined using FloJo software (Treestar Inc., Ashland, OR), and defined as the % of cells exhibiting relative TMRE fluorescence above 10^3^ arbitrary fluorescence units, as previously described (Burman et al., 2012). All flow cytometry measurements were performed on a Becton-Dickinson LSR II flow cytometer.

### Blue native gel electrophoresis and in-gel activity staining

Assays were carried out using mitochondrial preparations from 14-day-old flies as previously described (Suthammarak et al., 2009; Wittig et al., 2006). All reagents were purchased from Sigma.

### Western blots

14-day-old whole flies were flash-frozen in liquid nitrogen and homogenized with a pestle in 2× lysis buffer (50 mM TRIS, pH 8.0; 300 mM NaCl; 2 mM EDTA; 1% SDS; 2% Triton X-100) with protease inhibitor cocktail (Sigma). Homogenates were centrifuged at 21,000 ***g*** for 5 minutes, and the supernatant subjected to western blot processing and analysis. Blots were labeled with monoclonal antibodies to actin (Millipore #MAB1501) diluted 1/25,000, the β-subunit of ATP synthase (Invitrogen #A21351) diluted 1/1500, the NDUFS3 subunit of complex I (Abcam #17D95) diluted 1/800, and an anti-HNE fluorophore antibody (Calbiochem 393206) diluted 1/2500. This antibody detects a stable, crosslinking product between two lysyl residues and 4-HNE: 2:1 Nα-acetyllysine-HNE fluorophore. The antigen is taken as a proxy for accumulated 4-HNE damage, or accumulated ROS damage in general (Sun et al., 2014; Tsai et al., 1998). All reagents were purchased from Sigma unless otherwise noted. Western blot band intensities were quantified using ImageJ gel analysis tools, and the intensities were expressed as the % of control values following normalization to actin (Abramoff et al., 2004). Complete western blots are shown in supplementary material Fig. S5 as a demonstration of samples run in parallel.

### Statistics

Unless otherwise stated, statistical significance tests were calculated using an unpaired two-tailed Student’s *t*-test.

## Supplementary Material

Supplementary Material
