# Supplementary material for: A Drosophila model of mitochondrial disease caused by a complex I mutation that uncouples proton pumping from electron transfer
Source: Dis Model Mech. 2014 Aug 1;7(10):1165–74. doi: 10.1242/dmm.015321 (PMC4174527; doi:10.1242/dmm.015321)
Supplement: Supplementary Material [file supp_7_10_1165__index.html]

Supplementary Material 

# A *Drosophila* model of mitochondrial disease caused by a complex I mutation that uncouples proton pumping from electron transfer

## DMM015321 Supplementary Material

**Files in this Data Supplement:**

- **Supplementary Material**
