# Supplementary material for: A Drosophila model of mitochondrial disease caused by a complex I mutation that uncouples proton pumping from electron transfer
Source: Dis Model Mech. 2014 Aug 1;7(10):1165–74. doi: 10.1242/dmm.015321 (PMC4174527; doi:10.1242/dmm.015321)
Supplement: Supplementary Material [file supp_015321_DMM015321.pdf]

Fig. S1. ND2 *sequence alignment.* Primary amino acid sequence alignment of ND2 from Homo sapiens, *Mus musculus, Danio rerio, Drosophila melanogaster, Caenorhabditis elegans* and *Escherichia coli* are depicted. Identical residues are colored red, and similar residues colored blue. The three amino acid deletion caused by the $ND2^{del1}$ mutation is indicated with a green bar (residues 186-188). An insertion of a serine residue caused by the $ND2^{ins1}$ mutation at position 189 is depicted by a purple bar, and the L71P missense substitution found in a human Leigh syndrome patient (Ugalde et al., 2007) is indicated by a red bar.

Fig. S2. *Rescue of stress-induced paralysis by overexpression of yeast Ndi1, and increased hyperoxia sensitivity in ND2 mutants.* (A) 20 to 23-day-old female *ND2* mutants expressing *Ndi1* under the control of a Hsp70-GAL4 driver, and age-matched sibling *ND2* mutants lacking *Ndi1* expression (control) were maintained at 25°C, and assayed for the length of time (seconds) that flies remained paralyzed following mechanical stress. Error bars represent standard error of the mean; n=3 independent groups of 15 individual animals. p=0.044; *=p<0.05. (B) Survival of *ND2* mutants and controls in 100% oxygen. Histograms depict the median survival of control (120 hours ± 1.533) and *ND2* mutants (96 hours ± 1.625) (n=4 groups of 25 animals for each genotype); ***=p<0.0001; Error bars represent the standard error of the mean.

Fig. S3. *ND2 mutants do not exhibit degenerative changes in muscle fibers***.** (A) Confocal images of indirect flight muscles from 40 to 42-day-old controls, 40 to 42-day-old *ND2* mutants, and 10-day-old *parkin* mutants immunostained for actin (Phalloidin (red)), cytochrome C (cyt C) (cyan), 4',6-diamidino-2-phenylindole (DAPI) (purple), and cleaved caspase 3 (CC3) (green). Scale bar= 20 μm (B) Quantification of the number of cleaved caspase 3 positive muscle cells from images of controls (n=11 animals), *ND2* mutants (n=8 animals), and *parkin* mutants (n=9 animals). p=0.049 for *parkin* mutants vs. w1118, and p=0.26 for *ND2* mutants vs. *w1118*. ns= not significant; *=p<0.05. Error bars represent standard error of the mean. (C) Quantification of the

average mitochondrial perimeter (µm) from images of control (n=5 animals; N=3369 mitochondria), *ND2* mutant (n=6 animals; N=2456 mitochondria) and *parkin* mutant (n=5 animals; N=880 mitochondria) muscle preparations. p=0.03 for *parkin* mutants vs. w1118, and p=0.61 for *ND2* mutants vs. *w1118*. ns= not significant; *=p<0.05. Error bars represent standard error of the mean.

Fig. S4: *Representative oxygen electrode traces obtained using mitochondria isolated from control or ND2 mutants*: Mitochondria were isolated from 14-day-old control or *ND2* mutants, and respiratory traces measured using a Clark-type electrode. 300 µg of protein was added to 0.5 ml respiration buffer at point A. At point B the complex I-linked substrates malate and pyruvate were added to an initial concentration of 10 and 20 mM, respectively. At point C, 90 pmol ADP was added to an initial concentration of 180 µM ("low ADP"). At point D, ADP was added to an initial concentration of 2 mM ("high ADP"). The rates of oxygen consumption were measured from the slopes of the oxygen traces for state 3, state 4, and maximal state 3, as indicated by the arrows. ADP/O ratios were measured as the ratio of the pmol ADP added at point C and the pmol oxygen atoms consumed between point C, and the transition between state 3 and state 4 respiration.

Fig. S5. *Decreased complex I abundance in ND2 mutants*. Western blot analysis of protein extracts from 14-day-old *ND2* mutants and controls reveals a decrease in the abundance of mitochondrial complex I, as indicated by (A, B) decreased levels of the complex I subunit NDUFS3, with (B) no decrease in the Complex Vβ subunit. For clarity, whole blots are shown with their respective molecular weight markers.

Fig. S6. *ND2 mutants do not exhibit changes in ROS-mediated protein damage*: A) Western blot analysis of protein extracts from *ND2* mutants and controls throughout an aging time course reveals no increase in ROS-mediated protein damage in *ND2* mutants relative to controls, as indicated by 4-HNE protein adduct levels at 1, 14, 21 or 35 days of age. B) Quantification of Western blot data from (A), expressed as the % of control values following normalization to actin. n=3 independent western blot experiments for each age group. Error bars represent standard error of the mean.

Figure S1

```
           Homo sapiens 1   ---------------------------------------MNPLAQPVIYSTIFAGTLITA 21
           Mus musculus 1   ---------------------------------------MNPITLAIIYFTIFLGPMITM 21
            Danio rerio 1   ---------------------------------------MNPYVLMILMSSLGLGTTLTF 21
Drosophila melanogaster 1   --------------------------------------MFNNSSKILFITIMIIGTLITV 22
 Caenorhabditis elegans 1   -------------------------------------------MIVFISLFTLFLTLLSI 17
       Escherichia coli 1   MDVTPLMRVDGFAMLYTGLVLLASLATCTFAYPWLEGYNDNKDEFYLLVLIAALGGILLA 60

           Homo sapiens 22  LSSHWFFTWVGLEMNMLAFIPVLTKKM-NPRSTEAAIKYFLTQATASMILLMAILFNNML 80
           Mus musculus 22  SSTNLMLMWVGLEFSLLAIIPMLINKK-NPRSTEAATKYFVTQATASMIILLAIVLNYKQ 80
            Danio rerio 22  SSSHWILAWMGLEINTLAIVPLMAQQH-HPRAVEATTKYFLIQAAAAMILFTSTTNAWI 80
Drosophila melanogaster 23  TSNSWLGAWMGLEINLLSFIPLLSDNN-NLMSTEASLKYFLTQVLASTVLLFSSILLMLK 81
 Caenorhabditis elegans 18  LTNNVIVWWS---IFLLMTVVFILLNK-SSKSYTSIFNYFVIQESLGLLFLLCSGG---- 69
       Escherichia coli 61  NANHLASLFLGIELISLPLFGLVGYAFRQKRSLEASIKYTILSAAASSFLLFGMALVYAQ 120

           Homo sapiens 81  SGQWTMTNTTNQYSS------------LMIMMAMAMKLGMAPFHFWVPEVTQGTPLTSGL 128
           Mus musculus 81  LGTWMFQQQTNSLIL------------NMTLMALSMKLGLAPFHFWLPEVTQGIPLHMGL 128
            Danio rerio 81  SGQWDVTGMPGPATS------------TAMMFALALKIGLAPMHFWLPEVLQGLDLLTGL 128
Drosophila melanogaster 82  N--NMNNEINESFTS------------MIIMSALLLKSGAAPFHFWFPNMMEGLTWMNAL 127
 Caenorhabditis elegans 69  ---------------------------LLQFFIILLKIGVAPLHFWIFNVTNNIFNYGLM 102
       Escherichia coli 121 SGDLSFVALGKNLGDGMLNEPLLLAGFGLMIVGLGFKLSLVPFHLWTPDVYQGAPAPVST 180

           Homo sapiens 129 LLLTWQKLAPISIMYQISPSL-------NVSLLLTLSILSIMAGSWGGLNQTQLRKILAY 181
           Mus musculus 129 ILLTWQKIAPLSILIQIYPLL-------NSTIILMLAITSIFMGAWGGLNQTQMRKIMAY 181
            Danio rerio 129 ILSTWQKLAPMALIIQTTQTT-------DPLILTSLGIASSLIGGWSGLNQTQLRKILAY 181
Drosophila melanogaster 128 MLMTWQKIAPLMLISYLN----------IKYLLLISVILSVIIGAIGGLNQTSLRKLMAF 177
 Caenorhabditis elegans 103 WFLTFQKLPFLTILLQIFWLS-------SVYILLFG----LLICYVQIFVMKSYKNLLII 151
       Escherichia coli 181 FLATASKIAIFGVVMRLFLYAPVGDSEAIRVVLAIIAFASIIFGNLMALSQTNIKRLLGY 240

           Homo sapiens 182 SSITHMGWMMAVLPYNPN------MTILNLTIYIILTTTAFLLL-----NLNS------- 223
           Mus musculus 182 SSIAHMGWMLAILPYNPS------LTLLNLMIYIILTVPMFMAL-----MLNS------- 223
            Danio rerio 182 SSIAHMGWMIIVIQYAPQ------LTLIALGTYIFMTSAAFLTL-----KVLS------- 223
Drosophila melanogaster 178 SSINHLGWMLSSLMISES------IWLILFFFYSFLSFVLTFMF-----NIFK------- 219
 Caenorhabditis elegans 152 SSTESFNWIVLGVFFSMFN-------TFYLFIYYFVL----------------------- 181
       Escherichia coli 241 SSISHLGYLLVALIALQTGEMSMEAVGVYLAGYLFSSLGAFGVV-----SLMSSPYRGPD 295

           Homo sapiens 224 STTTLLLSRTWNKLTWLTPLIPSTFLSLGGLPPLTGFLPKWAIIEEFTKNNSLIIPTIMA 283
           Mus musculus 224 SMTINSISLLWNKTPAMLTVISLMLLSLGGLPPLTGFLPKWIIITELMKNNCLIMATLMA 283
            Danio rerio 224 ATKINTLTTTWPKSPILAAIATLVMLSLGGLPPLTGFMPKWLILQELTKQDLPATATIMA 283
Drosophila melanogaster 220 LFHLNQLFSWFVNSKILKFTLFMNFLSLGGLPPFLGFLPKWLVIQQLTLCNQYFMLTIMM 279
 Caenorhabditis elegans 181 ---MVLLISKFSKTSGYNFINWETTLVFLNIPFSVSFFVKIFSLSEIFKYDSFFTLFLLF 238
       Escherichia coli 296 ADSLFSYRGLFWHRPILAAVMTVMMLSLAGIPMTLGFIGKFYVLAVGVQAHLWWLVGAVV 355

           Homo sapiens 284 TITLLNLYFYLRLIYSTSITLLPMSN-NVKMKWQFEHTKPTPFLPTLIALTTLLLPISPF 342
           Mus musculus 284 MMALLNLFFYTRLIYSTSLTMFPTNN-NSKMMTHQTKTKPNPMFSTLAIMSTMTLPLAPQ 342
            Danio rerio 284 LTALLSLFFYLRLCHAMTLTTSPNTI-NSAPHWRVQTTQNSLPLTISVTVTMGLLPLTPA 342
Drosophila melanogaster 280 MSTLITLFFYLRICYSAFMMNYFEN--NWIMKMNMNSINYNMYMIMTFFSIFGLFLISLF 337
 Caenorhabditis elegans 239 TMFLSVLAFSFWLIN-------------LSMKNNEETSNNN---KMNYFIIFPLMVISII 282
       Escherichia coli 356 VGSAIGLYYYLRVAVSLYLHAPEQPGRDAPSNWQYSAGGIVVLISALLVLVLGVWPQPLI 415

           Homo sapiens 343 MLMIL----- 347
           Mus musculus 343 LII------- 345
            Danio rerio 343 ILMLTT---- 348
Drosophila melanogaster 338 YFMF------ 341
 Caenorhabditis elegans 282 ---------- 282
       Escherichia coli 416 SIVRLAMPLM 425
```

Figure S2

A

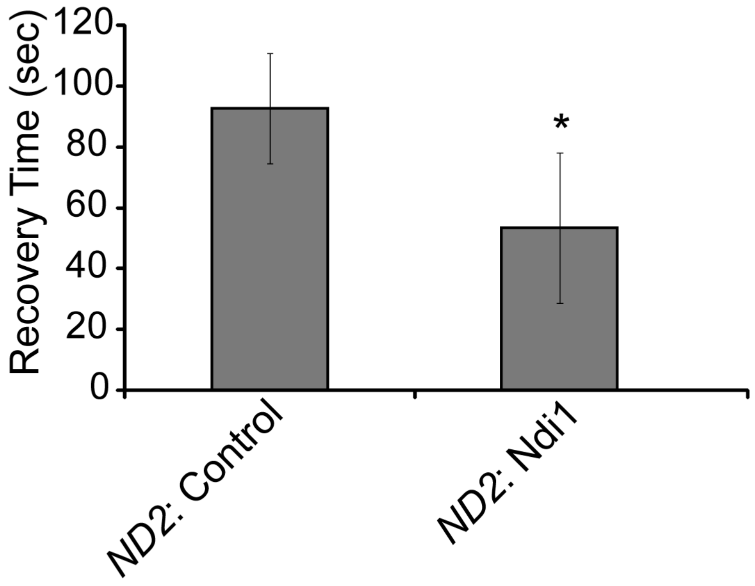

Recovery Time (sec)
120
100
80
60
40
20
0
*
ND2: Control
ND2: Ndi1

B

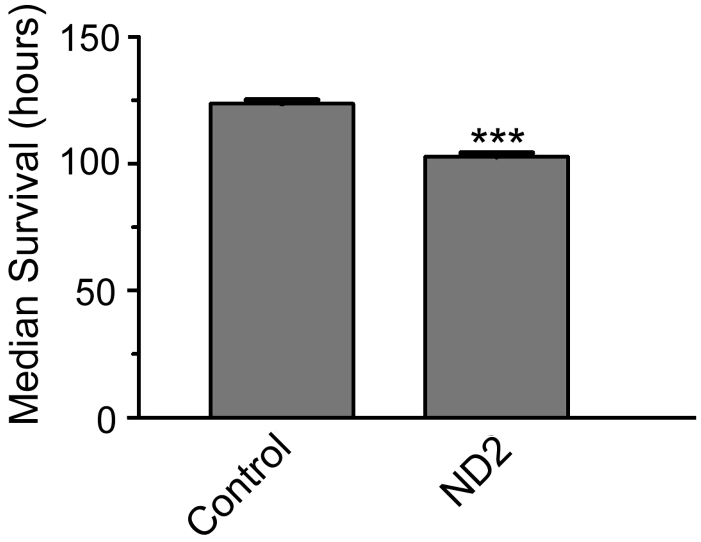

Median Survival (hours)
150
100
50
0
***
Control
ND2

Figure S3

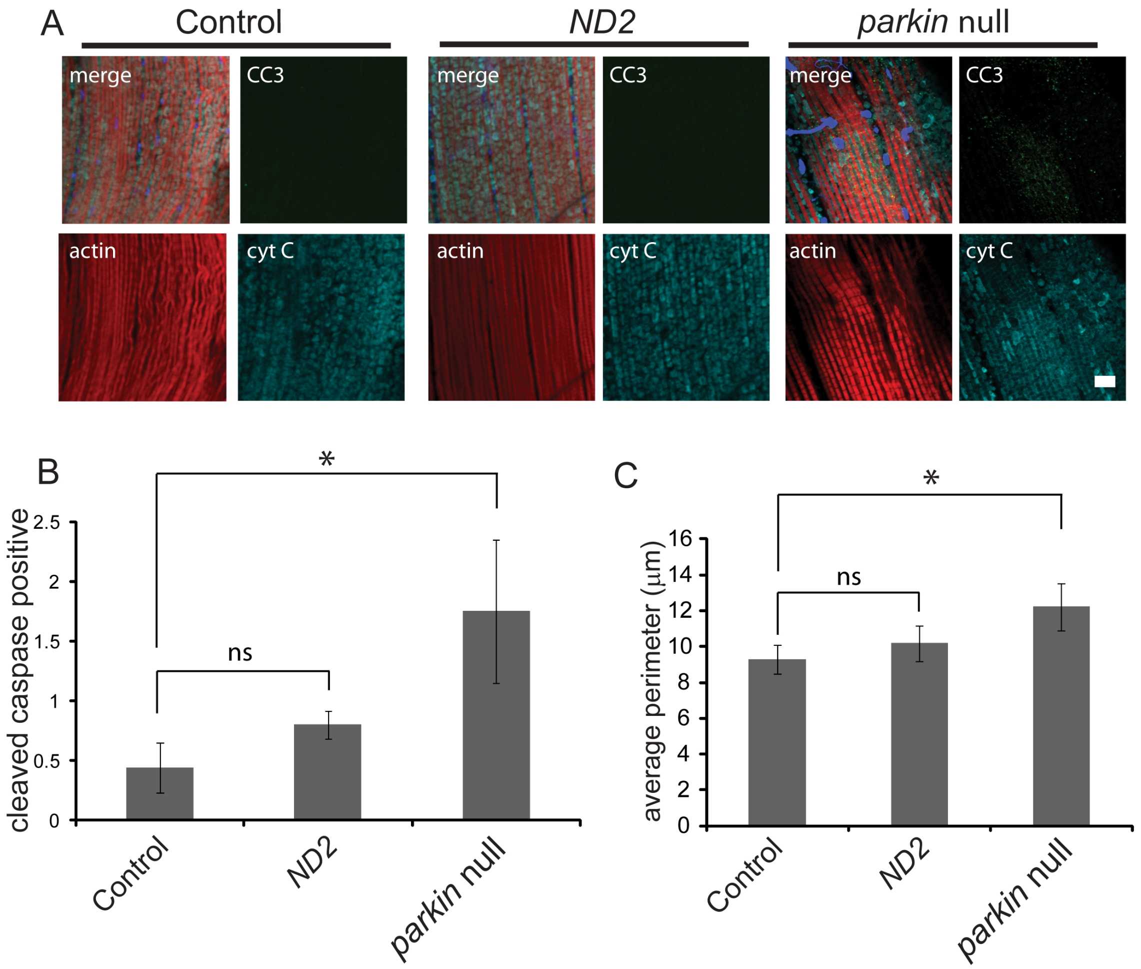
A
Control
ND2
parkin null
merge
CC3
actin
cyt C
B
*
ns
cleaved caspase positive
0
0.5
1
1.5
2
2.5
Control
ND2
parkin null
C
*
ns
average perimeter (μm)
0
2
4
6
8
10
12
14
16
Control
ND2
parkin null

Figure S4

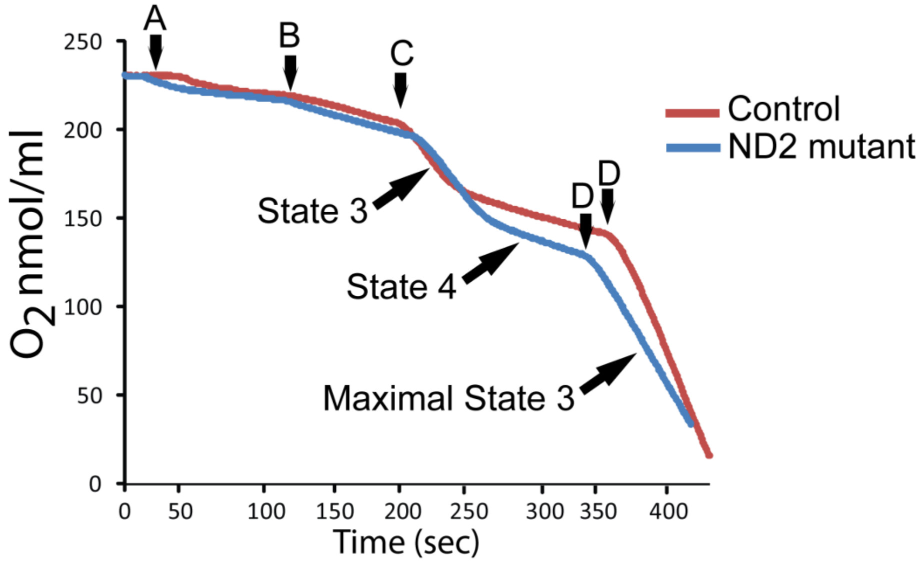
A
B
C
D
D
250
200
150
100
50
0
O2 nmol/ml
0
50
100
150
200
250
300
350
400
Time (sec)
Control
ND2 mutant
State 3
State 4
Maximal State 3

Figure S5

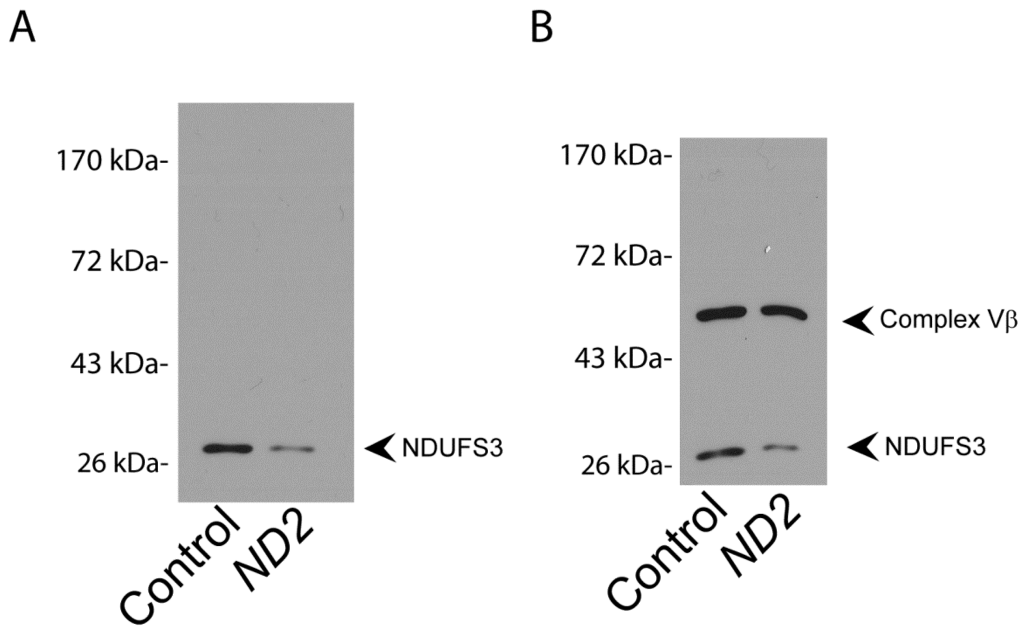
A
170 kDa-
72 kDa-
43 kDa-
26 kDa-
NDUFS3
Control
ND2
B
170 kDa-
72 kDa-
43 kDa-
26 kDa-
Complex Vβ
NDUFS3
Control
ND2

Figure S6

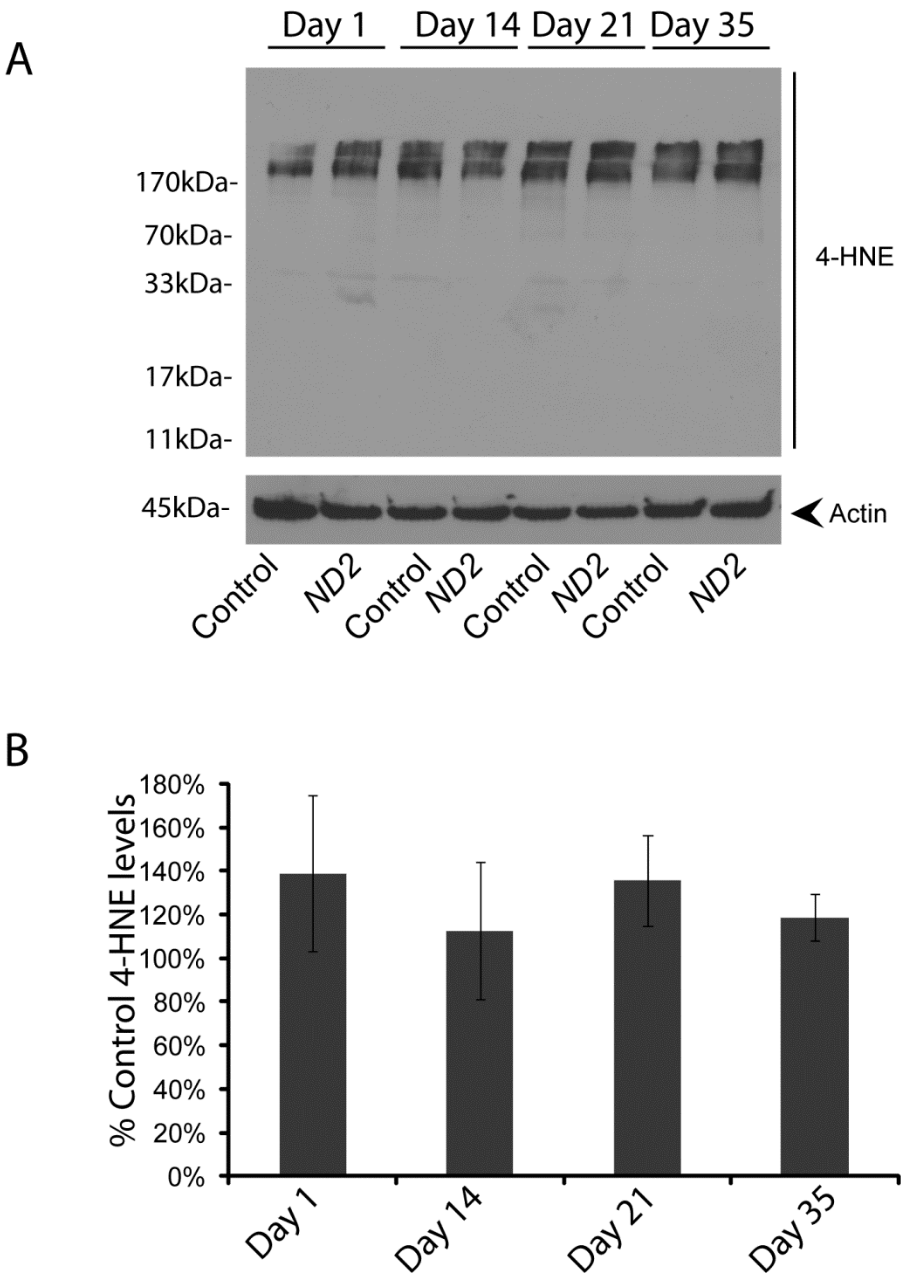
A
Day 1
Day 14
Day 21
Day 35
170kDa-
70kDa-
33kDa-
17kDa-
11kDa-
4-HNE
45kDa-
Actin
Control
ND2
Control
ND2
Control
ND2
Control
ND2
B
% Control 4-HNE levels
180%
160%
140%
120%
100%
80%
60%
40%
20%
0%
Day 1
Day 14
Day 21
Day 35

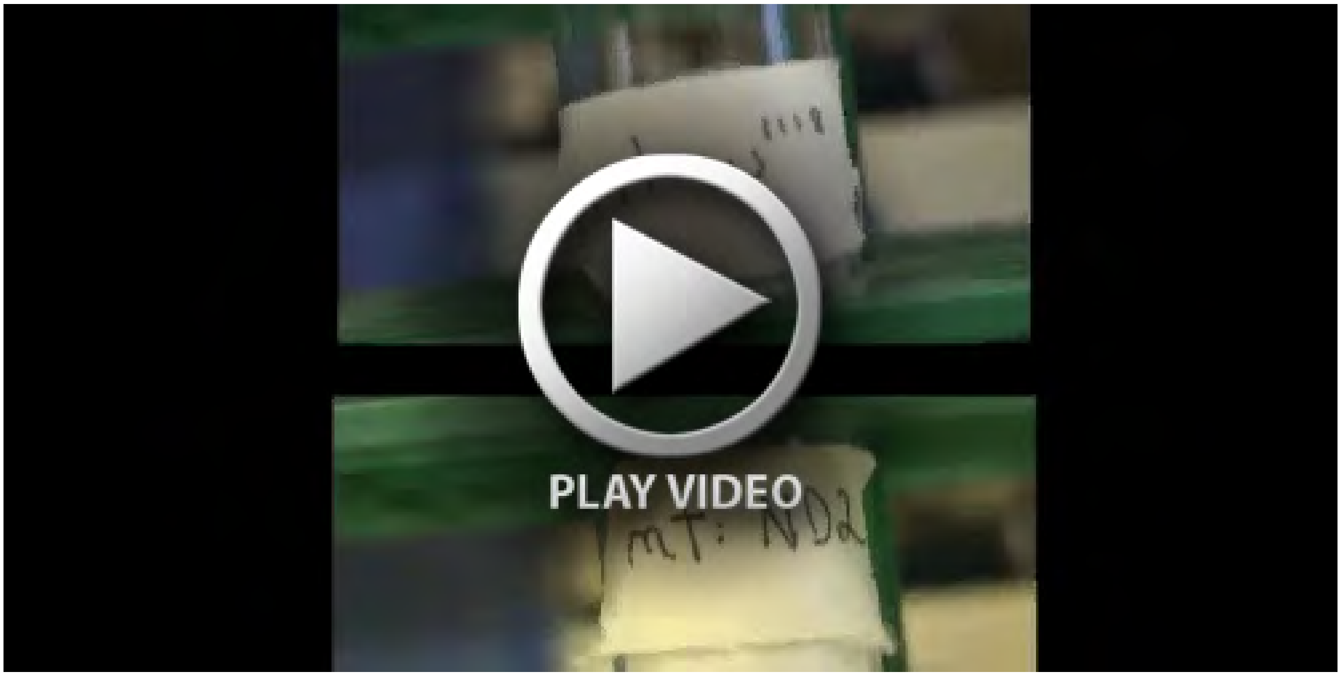

Movie S1. *Mechanical stress-induced paralysis of ND2 mutants.* 21-day-old female *ND2* mutants and controls were vortexed in inverted vials containing cotton stoppers for 10 seconds, and then filmed in real time. Controls (upper frame) were repeatedly vortexed throughout the movie, but displayed no movement defects. However, *ND2* mutants (lower frame) remained paralyzed for >50 seconds before righting themselves.
